# Dynamic multi-outcome prediction after injury: Applying adaptive machine learning for precision medicine in trauma

**DOI:** 10.1371/journal.pone.0213836

**Published:** 2019-04-10

**Authors:** S. Ariane Christie, Amanda S. Conroy, Rachael A. Callcut, Alan E. Hubbard, Mitchell J. Cohen

**Affiliations:** 1 Department of Surgery, Zuckerberg San Francisco General Hospital and Trauma Center and the University of California, San Francisco; San Francisco, California, United States of America; 2 Department of Biostatistics, University of California, Berkeley School of Public Health; Berkeley, California, United States of America; 3 Denver Health Medical Center and the University of Colorado; Denver, Colorado, United States of America; National Yang-Ming University, TAIWAN

## Abstract

**Objective:**

Machine learning techniques have demonstrated superior discrimination compared to conventional statistical approaches in predicting trauma death. The objective of this study is to evaluate whether machine learning algorithms can be used to assess risk and dynamically identify patient-specific modifiable factors critical to patient trajectory for multiple key outcomes after severe injury.

**Methods:**

SuperLearner, an ensemble machine-learning algorithm, was applied to prospective observational cohort data from 1494 critically-injured patients. Over 1000 agnostic predictors were used to generate prediction models from multiple candidate learners for outcomes of interest at serial time points post-injury. Model accuracy was estimated using cross-validation and area under the curve was compared to select among predictors. Clinical variables responsible for driving outcomes were estimated at each time point.

**Results:**

SuperLearner fits demonstrated excellent cross-validated prediction of death (overall AUC 0.94–0.97), multi-organ failure (overall AUC 0.84–0.90), and transfusion (overall AUC 0.87–0.9) across multiple post-injury time points, and good prediction of Acute Respiratory Distress Syndrome (overall AUC 0.84–0.89) and venous thromboembolism (overall AUC 0.73–0.83). Outcomes with inferior data quality included coagulopathic trajectory (AUC 0.48–0.88). Key clinical predictors evolved over the post-injury timecourse and included both anticipated and unexpected variables. Non-random missingness of data was identified as a predictor of multiple outcomes over time.

**Conclusions:**

Machine learning algorithms can be used to generate dynamic prediction after injury while avoiding the risk of over- and under-fitting inherent in ad hoc statistical approaches. SuperLearner prediction after injury demonstrates promise as an adaptable means of helping clinicians integrate voluminous, evolving data on severely-injured patients into real-time, dynamic decision-making support.

## Introduction

Modern trauma and critical care medicine is characterized by voluminous data [1]. Advanced monitoring systems reflect the physiologic state of critically injured patients in real time making it possible to access unprecedented amounts of patient-level data [2]. Advanced analytics, including new types of machine learning, can be utilized to extract value from this voluminous data for real time prediction and ultimately could lead to bedside precision-medicine-based decision making [3].

Although a few academic institutions have pioneered data-mining applications in large clinical databases of critically-ill patients [2, 4–6], general trauma and surgical communities have not yet fully embraced the potential of data mining. In an era when advanced analytics are ubiquitous on our smartphones, many providers still rely on clinical gestalt and/or scoring algorithms to guide decision making. Scoring algorithms tend to be highly constrained to maintain simplicity. While often published with good initial sensitivity and specificity many cannot be validated in subgroups, larger datasets, or fail over time as they are not adaptable to the changing clinical context [7–10]. Clinical acumen will always be an essential component of critical care, but even seasoned clinicians cannot systematically integrate all the information available on critically injured patients throughout their hospital course, nor is it possible to anticipate patterns only apparent at the aggregate level [11].

**Fig 1:**
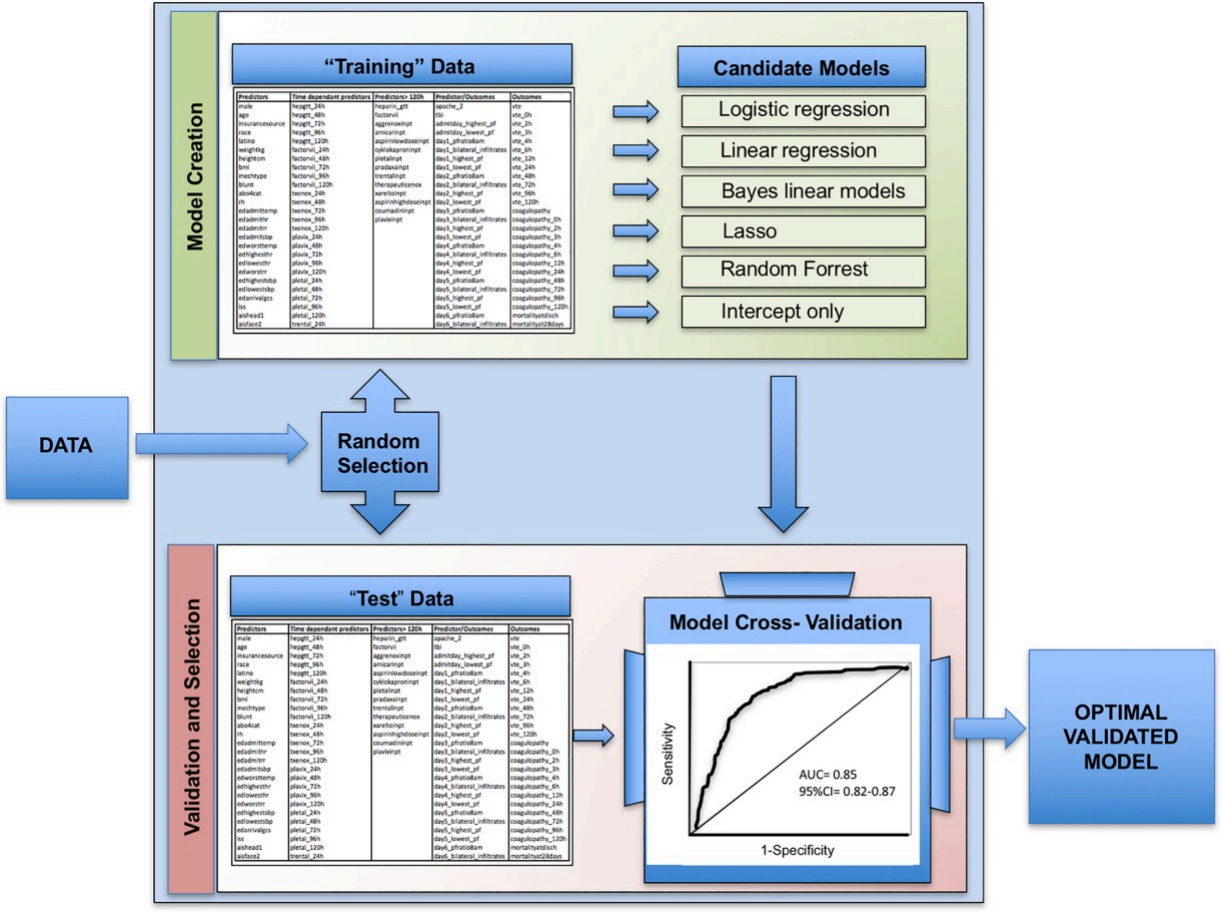
SuperLearner prediction. A simplified schematic of the SuperLearner selection of optimized models from large datasets using an unbiased approach of potential predictors. AUC, Area Under the Curve; CI, Confidence Interval. Candidate models listed are intended to be examples and do not represent all models used in this analysis.

In other disciplines, machine learning analytics have been utilized to harness the power of data. These methods evaluate potential predictors in an agnostic fashion and model complex and unanticipated relationships between the hundreds or thousands of variables that contribute to a single outcome [11]. SuperLearner, an ensemble machine learning algorithm, is one such technique (Fig 1). Rather than pre-specifying a single statistical approach, SuperLearner simultaneously investigates multiple algorithms ranging from simple logistic regression to highly complex machine learning (e.g., neural nets) in order to optimally predict outcomes of interest from complex datasets [12]. Embedded cross-validation reduces the risk of over-fitting seen with many traditional statistical approaches [13]. Additionally, SuperLearner is readily modifiable suggesting that it may be well-suited for tracking dynamic prediction over time. Identification of the clinical variables that drive prediction at critical timepoints may reveal targets for altering patient trajectory after severe injury [14, 15].

Prior application has established proof-of-concept that SuperLearner outperforms conventional statistical approaches to mortality prediction after severe injury, and is able generate high-fidelity prediction over the post-injury timecourse [13, 16]. Influential predictors of trauma mortality, termed “Variable Importance Measures,” likely evolve with time [16]. However, these studies generated prediction and variable importance from a limited number of pre-specified candidate variables; SuperLearner performance using a large uncurated predictor set remains unknown. Additionally, prior applications have largely been limited to prediction of trauma mortality.

The objective of this study was to assess the capacity of a machine learning algorithm to predict multiple outcomes of interest over time in a critically-injured patient population using a large number of candidate predictors. We demonstrate that SuperLearner is capable of generating dynamic prediction for many outcomes, but also that even with advanced machine learning approaches, prediction capacity depends on data quality. We further demonstrate that variable importance evolves dynamically with different, often unanticipated, variables driving prediction at progressive points in the post-injury timecourse.

## Materials and methods

### Cohort description

The Activation of Coagulation and Inflammation in Trauma study (ACIT) (S1 dataset) was a previously-described single-center prospective cohort study which followed severely injured trauma patients from emergency department admission through the first 28 days of hospitalization or death [17, 18]. Between February 2005 and April 2015, 1494 trauma patients meeting criteria for highest triage activation level were enrolled into the study. Exclusion criteria included patient age less than 15 years, pregnancy, incarceration, and transfer from outside hospital. Written consent was obtained from enrolled patients or their families or, rarely, in certain circumstances where these could not be obtained, a waiver of consent was utilized [17, 18]. The study was carried out with the approval of the University of California Institutional Review Board (reference number 10–04417).

The data set included 2,397 variables on 1,494 patients. Of these, 1,880 variables had sufficient variability to potentially affect prediction and were used in model generation. For the purposes of this study sufficient variability was considered to be a minimum of 50 cases at each level for a given variable (i.e. for a variable assessing tobacco use, there would need to be at least 50 tobacco users and 50 non-tobacco users for the variable to be included as a potential predictor) as fewer instances than this would be unlikely to have suffient power to affect prediction.

Baseline patient data included patient demographics, past medical history, substance use, and injury characteristics. Physiologic variables tracked from emergency admission to ICU discharge included: vital signs, laboratory monitoring including coagulation and inflammation markers, ventilator parameters, input/output data, and all fluid, colloid, blood product and medication administration. Embedded injury scoring including Injury Severity Score [19], Glasgow Coma Scale [20], Denver Post-injury Multiple Organ Failure Score [21] and APACHE II [22], all of which were assessed daily. Outcomes data included blood product transfusion, massive transfusion (>10L in 24 hours or equivalent), total ventilator days, critical care days, length of hospitalization, development of nosocomial infection, venous thromboembolic events, acute respiratory distress syndrome (ARDS) [23], coagulopathy (International Normalized Ratio>1.4 and/ or Partial Thromboplastin Time ≥35) and coagulopathic trajectory (the trend in coagulopathy), organ injury and mortality.

Clinical, laboratory and interval outcomes data were collected at admission, 2, 3, 4, 6, 12, 24, 48, 72, 96, and 120 hours after injury. All prediction models were generated in time-dependent formats, using data available up to a specified interval cutoff to predict outcomes at subsequent time periods. For a list of predictor set times and outcomes, please see Table 1.

**Table 1:** Interval prediction sets by outcome investigated.

Outcomes of Interest	2h	3h	4h	6h	12h	24h	48h	72h	96h	120h
**Death**	**X** ^*c*^	**X**	**X**	*	**X**	**X**	**X**	**X**	*	*
**Venous Thromboembolism**	*	*	*	*	*	*	*	*	**X**	**X**
**Multi-Organ Failure**						**X**	**X**	**X**	**X**	**X**
**Acute Respiratory Distress Syndrome**						**X**	**X**	**X**	**X**	**X**
**Coagulopathic Trajectory (PT** ^*a*^**)**	**X**	**X**	**X**	**X**	**X**	**X**	**X**	**X**	**X**	**X**
**Coagulopathic trajectory (PTT**^*b*^**)**	*	*	**X**	**X**	**X**	**X**	**X**	**X**	**X**	**X**
**Transfusion**				**X**	*	**X**	**X**	**X**		
**Massive Transfusion**				**X**	*	*	*	*		


^*a*^ PT, prothrombin time

^*b*^ PTT, partial thromboplastin time

^*c*^ X indicates SuperLearner prediction supported at this time interval

_*_ indicates that there were insufficient events to support SuperLearner prediction at this time interval

### SuperLearner prediction

An ensemble machine learning algorithm, SuperLearner, was applied to this dataset to generate prediction of each outcome of interest at progressive timepoints. SuperLearner uses embedded cross-validation to optimize prediction. Although more extensively described elsewhere, cross-validation essentially involves separating the data into a “training set” of data used to generate a particular model and validating the analysis on a “hidden” test set not seen by the algorithm during model generation. SuperLearner uses V-fold cross-validation, where the candidate learners are fit on the V training samples, and these used to predict the outcomes on the corresponding validation sample. The final ensemble learner is then fit as a convex combination of the individual learners. To examine the performance of SuperLearner, the entire procedure was nested in another V-fold cross-validation, where the SuperLearner was fit on the V training samples (this itself involves cross-validation within the training sample) and the fit assessed on the left out “testing” samples to derive an unbiased cross-validated AUC fit. The SuperLearner does a meta-level of algorithm selection by using non-negative least squares such that the final fit only weights a subset of algorithms, the others not contributing to final fit.

We utilized the following candidate prediction algorithms: logistic and linear regression, generalized additive models with various levels of smoothing [24], random forest [25], lasso [26] and systems based on sieves of parametric models (e.g. polyclass). Inference for the performance of each model was estimated using cross-validated risk [27]. All outcomes were treated as binary and area under the curve (AUC) of a receiver-operator curve (ROC) was used to select learner combinations.

In order to better explain the model generation procedure using SuperLearner, consider death as a single example of an outcome of interest. The dataset was structured in time increments (admission to 2 hours after injury, 2 to 3 hours, 3 to 4 hours, 4 to 6 hours and etc.) including the indicator of whether the patient died within each interval. Using all diagnostic variables available to clinicians at a given timepoint (for example, at admission), SuperLearner was employed to estimate (predict) among those still alive, the instantaneous probability (hazard) of death at each subsequent interval of time, conditional on both values of the predictor as well as the history of measurements.

Data with missing outcomes was dropped from the analysis. One goal of the study was to interpret patterns of missingness among predictors, given that for real-world trauma data one cannot expect all variables will be recorded. To evaluate the role of missing data, first, all nominal variables were converted to the appropriate dummy variables. Then, we constructed a new set of basic functions for every variable with any missing observations, specifically we made, for variable X, an indicator that X is observed, say Δ, that is one if the variable is observed, 0 otherwise and then a variable Δ*X that is the actual observed value if it is observed and 0 otherwise. In this manner, the procedure can learn both from the missing values and from the observed values.

For two high-performance outcomes, we applied a variable importance measure available in the ensemble learning method random forest. Compared to more parametric forms tested, random forest was given a much greater weight in the ensemble for all of our prediction models suggesting that random forest would provide the most robust estimate of the relative variable importance. Briefly, the random forest algorithm estimates the importance of a variable by looking at how much prediction error increases when data for that variable is permuted while all others are left unchanged. The basic variable importance looks at the change in misclassification rate in the original data versus the particular variable being permuted (and thus made independent of the outcome). Thus, it is a relatively intuitive measure of importance, targeting how important the variable is for accurately predicting the outcome given the other variables. which estimates the relative loss to prediction accuracy by removing the variable [25, 28]. We measured random forest variable importance by the change in the Gini measure of impurity, which has been found to give back more robust results in other settings. For the purposes of this study, “high-performance outcomes” were considered to be those with very good or excellent model cross-validated prediction as indicated by area under the curve> 85%. Variables with the largest difference suggest highly impactful variables on the model outcome. Influential predictors for these outcomes were compared over post-injury timepoints.

## Results

### Cohort demographics

Between February 2005 and May 2015 1,494 patients were enrolled in our cohort (Table 2) This group represented a standard severely-injured trauma population and was predominantly young (median age 36) and male (82%), with 57% of injuries attributable to blunt mechanisms. Forty-seven percent of patients had an Injury Severity Score greater than 15. Approximately 15% of patients were coagulopathic at admission and 17% received massive transfusion within the first 72 hours of admission. Sixteen percent developed ARDS and 8.6% developed multi-organ failure (MOF) during admission. Mortality at 28 days was 17.4%.

**Table 2:** Demographics, selected clinical variables and outcomes of the activation of coagulation and inflammation in trauma cohort.

	Percentage/Mean ± SD *or* Median (IQR)
**Age (years)**	**36** (25–52)
**Male (%)**	**82** ± 38
**Body Mass Index (kg/m**^**2**^**)**	**26.8** ± 5.26
**Injury Severity Score**	**14** (4–27)
**Blunt Mechanism (%)**	**57** ± 49
**Traumatic Brain Injury (%)**	**37** ± 48
**Prehospital Crystalloid (mL)**	**50** (0–250)
**Temperature**^*a*^ **(°C)**	**36.5** (35.7–36.8)
**Base Deficit**^*a*^ **(mEq/L)**	**-3.1** (-7.5–0.5)
**Platelet Count**^*a*^ **(1000/μL)**	**273** ± 86
**Fibrinogen**^*a*^ **(mg/dL)**	**220** (165–283)
**Protein C**^*a*^ **(% activity)**	**88** (71–107)
**D-dimer**^*a*^ **(μg/dL)**	**1.77** (0.39–6.47)
**Transfused between 0-72h (%)**	**42** ± 50
**Intensive Care Unit Days**^*b*^	**4.93** ± 0.52)
**Ventilator Free Days**^*b*^	**26** (11–28)
**Venous Thromboembolic Embolism (%)**	**5** ± 22
**Mortality at 24h (%)**	**9** ± 28


^*a*^ Variable measured at admission

^*b*^ During first 28 days of admission

### Overall prediction

**Fig 2:**
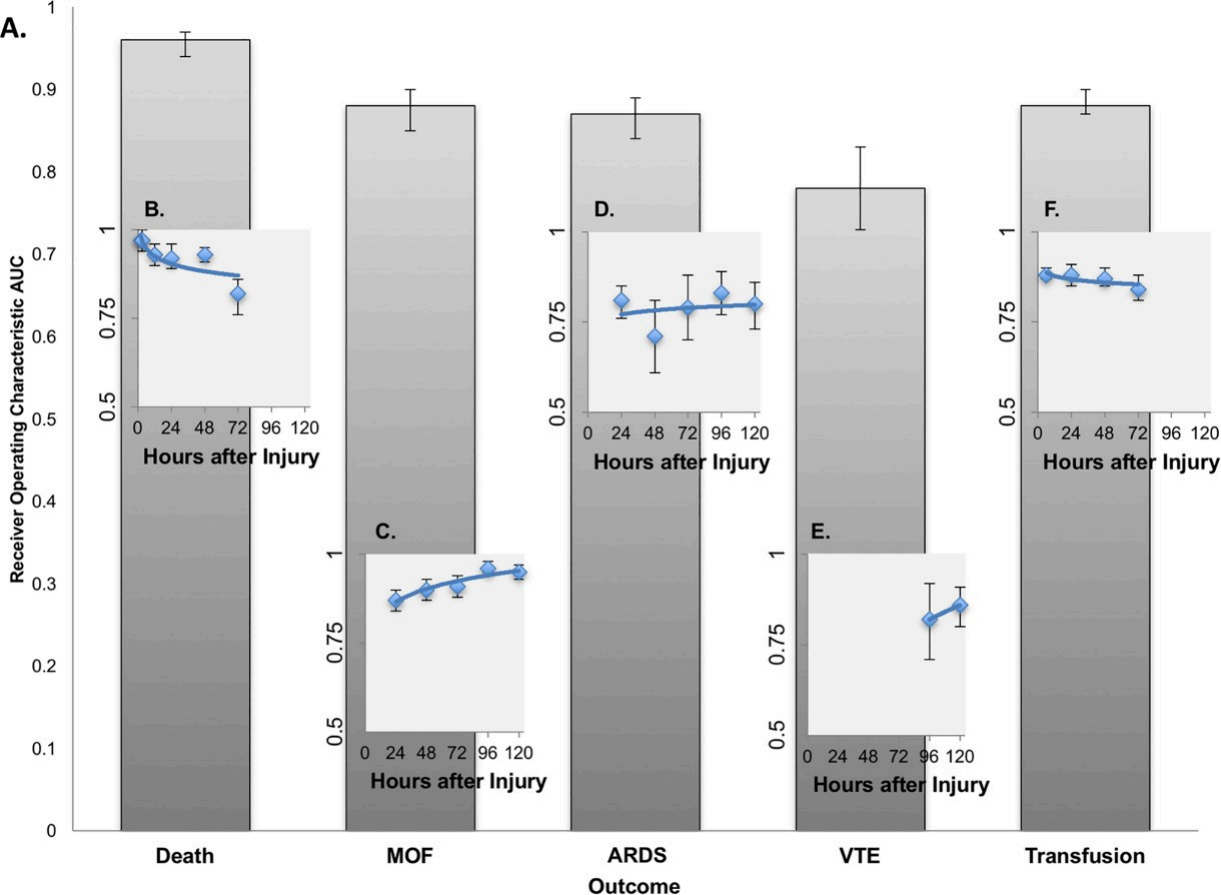
Overall and interval prediction of key outcomes after injury using SuperLearner. A. Overall SuperLearner Prediction of Key Outcomes after Injury using Admission Data B. Interval SuperLearner Prediction of Death after Injury C. Interval SuperLearner Prediction of Multi-Organ Failure by after Injury D. Interval SuperLearner Prediction of Acute Respiratory Distress Syndrome by Interval after Injury E. Interval SuperLearner Prediction of Venous Thromboembolic Events by Interval after Injury F. Interval SuperLearner Prediction of Transfusion Requirement by Interval after Injury. ROC, Receiver Operating Characteristic; AUC, Area Under the Curve; MOF, Multi-Organ Failure; ARDS, Acute Respiratory Distress Syndrome; VTE, Venous Thromboembolic Event.

Prediction of overall outcomes based on only variables available at admission provided robust discrimination of multiple critical outcomes, including death (AUC 0.96, 0.94–0.97), MOF (AUC 0.88, 0.84–0.9), ARDS (AUC 0.87, 0.84–0.89), and early blood transfusion (AUC 0.88, 0.87–0.90) (Fig 2).

### Interval prediction

Variability in the discrimination of SuperLearner prediction of critical outcomes was seen over time, as data reflecting and each patient’s updated physiology and trajectory accumulated (Fig 2).

#### Death

Discrimination of death by 2 hours based on admission data was nearly perfect (AUC 0.97, 0.96–0.98). After this early interval, our capacity to predict mortality remained excellent (AUC 0.92–0.97) but decreased as patients progressed further from the injury event. Prediction of death at 72 hours based on 48 hour predictors was the least robust (AUC 0.82, 0.76–0.88) (Fig 2).

#### Multi-organ failure

Early discrimination of MOF at 2 hours based on admission predictors was also robust (AUC 0.87) but progressively improved as longitudinal predictors reflecting updated physiology and trajectory were added to the modeling framework (AUC 0.9–0.96) (Fig 2).

#### Venous thromboembolic events

Venous thromboembolic events (VTE) did not occur with sufficient frequency before 72 hours to support SuperLearner prediction. VTE at 96 hours based on 72-hour data demonstrated fair to good discrimination (AUC 0.82) but improved substantially by 120h as SuperLearner was able to build from additional events (AUC 0.86).

#### Acute respiratory distress syndrome

Interval based discrimination of ARDS throughout all timepoints was fair to good (AUC 0.71–0.83), with wide confidence interval margins, but at no single timepoint was interval prediction able to achieve the performance of the overall model based on admission predictors. This suggests that while admission data were sufficient to generate robust prediction of *which* patients were likely to develop ARDS post injury, it was not possible to predict *when* ARDS was imminent even with progressive addition of longitudinal predictors (Fig 2).

#### Transfusion

Interval discrimination of blood transfusion remained stably robust throughout the first 72 hours (AUC 0.84–0.88), although there was insufficient data for SuperLearner to generate prediction of transfusion in the 6 or 12-hour interval (Fig 2).

#### Coagulopathic trajectory

SuperLearner was not able to generate robust prediction models for coagulopathic trajectory at any interval between 2 and 120 hours after injury. Prediction was poor for coagulopathic trajectory based on both prothrombin time (AUC 0.45–0.73) and partial thromboplastic time (AUC 0.48–0.74).

### Prediction variable importance measures

**Fig 3:**
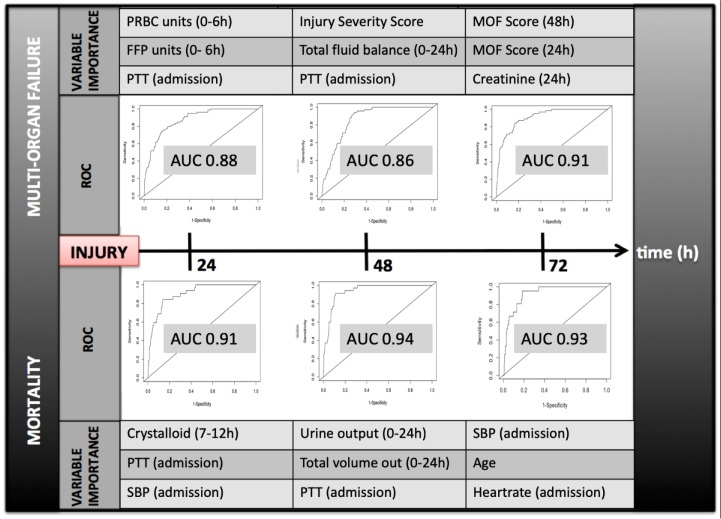
Variable importance by random forest for multi-organ failure and death at 24, 48, and 72 hours after injury. For each timepoint and outcome of interest the top three variables driving prediction are reported. h, hours after presentation; PRBC, packed red blood cells transfused within the first 6 hours; FFP, fresh frozen plasma transfused within the first 6 hours; MOF, multi-organ failure; PPT, partial thromboplastin time ROC, receiver operating characteristic curve, AUC, Area Under the Curve; SBP, Systolic blood pressure.

For two outcomes of interest with high performing prediction models, random forest was used to identify key predictors for the 24 hour, 48 hour, and 72 hour timepoints (Fig 3). For both death and MOF, variable importance evolved between timepoints, with different predictors possessing greater weight in predicting outcomes at each interval. Non-random missingness was found repeatedly to contribute substantially to prediction but was not a top variable importance measure for these outcomes.

## Discussion

In this paper, we demonstrate that machine learning can be utilized to generate dynamic prediction of multiple critical outcomes after injury. SuperLearner was able to establish near-perfect discrimination for death and MOF across the post-injury timecourse using large uncurated sets of potential predictors. In instances where prediction was less robust, SuperLearner provided a metric of the optimal prediction achievable based on the quality and volume of available data. Finally, our findings highlight the evolution of prediction drivers for key outcomes as patients move through early post-injury care. Dynamic variable importance may explain why static algorithms which rely on a few fixed predictors lead to overfit and less useful prediction over time. Notably this pattern mimics the way in which seasoned clinicians already think about the trajectory of patients with the “most important” clinical information differing from minute to minute and hour to hour.

Assessment of risk and prediction of patient trajectory has been an ongoing goal following critical injury. Over time different techniques purporting to distill complex data into algorithms capable of predicting critical outcomes like death have been used to guide management and as metrics to measure trauma center performance [19, 29–35]. However, prediction algorithms using conventional statistical approaches have demonstrated a variety of limitations and often fail to perform as well as their initial pilot AUC curves would anticipate [36–40]. One important reason for this is that these algorithms only consider a very small set of models, largely constructed using variables clinicians already think are important resulting in inherently biased models which leave lots of information on the table. Traditional statistical methods cannot sufficiently adjust for the complex, often nonlinear, relationships of patient outcomes and the large number of variables measured densely over time [41].

Machine learning has offered one potential solution to many of these challenges and prior studies have demonstrated that algorithms such as SuperLearner are capable of generating superior discrimination of trauma death comparted to conventional statistical approaches [13, 16]. The foundational paradigm of data mining is to treat data in an agnostic fashion, allowing organic relationships to emerge rather than imposing the constraints of arbitrary parametric models [41]. This is not immediately comfortable for most medical investigators, who are disquieted by black box approaches and want easily interpretable results. However, theoretical results and demonstration studies have established that SuperLearner performs as well as any candidate learner in the set being considered, and that it is not possible to try too many learners as cross-validation prevents over-fitting [13, 42]. The more candidate learners, the greater chance that the best provide optimal prediction for the particular distribution [12, 15]. Here, we present the first data demonstrating that we can apply SuperLearner to a much larger, uncurated predictor pool to generate dynamic prediction of multiple critical outcomes after injury. This is critical to enhance usability of these methods for clinicians who may not have time to carefully pre-select potential predictors and to allow for data-driven detection of patterns which might not be expected based on clinical gestalt.

### Predictive capacity

In this investigation, where the data was of sufficient volume and quality, SuperLearner demonstrated excellent discrimination over time. For some outcomes, including MOF, interval prediction improved as predictors reflective of updated physiology and clinical trajectory were added. However, it is equally important to note where SuperLearner’s ability to predict outcomes failed to improve because these patterns have important implications for understanding the correlative power and limitations of data. Prediction may worsen over time because the outcome becomes more infrequent later in the clinical course. In other cases, a particular outcome may be closely correlated (or caused) by the available variables early after injury but later may depend on unidentified influential variables not included in the sample. Knowledge of the patterns of predictive capacity can lend insight into when it is most efficacious to employ or rely upon prediction algorithms.

At timepoints and for outcomes where prediction performance was marginal or poor, SuperLearner provided a metric of where data quantity or quality was insufficient to support reliable prediction. SuperLearner provides a ceiling in the performance one can expect from other algorithms that use the same data to construct prediction. If a simplified statistical algorithm is generated, it should be measured against the same cross-validated measure of performance (e.g., AUC) as that reached by SuperLearner. As embedded cross-validation protects against overfit, poor SuperLearner prediction heralds the need to identify and measure additional, potentially influential variables rather than to employ a different analytic methodology. Most clinicians recognize that simply introducing additional markers or monitors does not necessarily augment clinical understanding and at times can even confuse the situation. However, large datasets have been demonstrated to decrease the magnitude of spurious effect estimates [43] and machine learning algorithms like SuperLearner are ideally suited to help determine whether the addition of novel predictors (biomarkers, monitoring systems) add to the “signal” or just to the “noise” of model prediction.

### Variable importance

Equally important, the variables responsible for driving death and MOF varied over the early post-injury trajectory, bolstering evidence from prior studies which suggested that predictors of mortality likely evolve with time [13, 16]. In our study, agnostic use of all available predictors at each timepoint demonstrated that prediction drivers included variables that likely would have been anticipated by clinicians (such as markers of coagulopathy and transfusion requirements) as well as factors like total volume output, which were relatively unanticipated (Fig 3). Additionally, non-random missingness of specific variables was noted to drive prediction of outcome at several timepoints. While it is not possible to determine definitively why this occurs, we hypothesize that these variables may serve as surrogate indicators of clinical gestalt. For example, blood alcohol level may be missing in patients considered too ill by nursing staff to have received this routine lab test in the trauma bay. Further prospective investigation of the role and potential utility of indicators of non-random missingness is warranted.

### Limitations

While promising, this study has several limitations. First, the specific algorithms used within the SuperLearner model were no means exhaustive. As described above the combination selected encorporated very flexible and very smooth learners to cover a wide range of potential prediction functions. However, there are an increasing number of new learners every day, and inclusion of these learners may indeed improve prediction. We present here a proof of concept that machine learning can be used to generate effective dynamic prediction for key outcomes with messy, real-world trauma data and anticipate that improving prediction with the encorporation of new learners will remain an area of ongoing study. Additionally, as demonstrated above, the efficacy of both SuperLearner and variable importance identification rely directly on the quantity and quality of the data inputs. SuperLearner was not able to generate models for timepoints and outcomes where events or variability were too sparse (approximately less than 50 events). This is, in our estimation, the most important consideration of when machine learning applications are appropriate. Indeed, the use of these statistical techniques require investigators and clinicians to be diligent with regards rigorous database set-up and high-fidelity collection mechanisms to better potentiate the utility of these modalities.

### Potential applications

We anticipate that this methodology can be extended in several ways to directly improve the quality of trauma care. First, with sufficient data this system in contrast to “one-size-fits-all” approaches to trauma resuscitation [44–46], with sufficient data SuperLearner has the potential to be able to generate dynamic prediction for individual patients, and specifically to estimate the treatment effect of potential interventions in those patients for outcomes of interest. Further mapping of variable importance for key outcomes over time represents a fertile research opportunity for better understanding of the mechanistic processes underlying disease trajectory. Finally, these methods are readily scalable for cloud computing environments, and have the potential to be practically applied to streaming data from diverse patient populations for real-time decision support.

### Conclusions

Machine learning techniques can help clinicians rapidly integrate the voluminous, evolving data currently available on severely-injured patients into potentially real time, dynamic decision-making support. The use of cross-validation to select the optimal combination of models avoids both over-fitting by fitting noise instead of signal, but also under-fitting by fitting relatively simple, but poorly specified models. The application of SuperLearner to increasingly dense streaming patient data holds significant promise for advancing individualized precision medicine for trauma critical care.

## Supporting information

S1 Dataset This dataset contains the data from the Activation of Coagulation and Inflammation in Trauma study (ACIT) used to generated the prediction described in this manuscript. ACIT is a previously-described single-center prospective cohort study which followed severely injured trauma patients from emergency department admission through the first 28 days of hospitalization or death. Between February 2005 and April 2015, 1494 trauma patients meeting criteria for highest triage activation level were enrolled into the study.
(DTA)
